# Detecting Congestive Heart Failure by Extracting Multimodal Features and Employing Machine Learning Techniques

**DOI:** 10.1155/2020/4281243

**Published:** 2020-02-18

**Authors:** Lal Hussain, Imtiaz Ahmed Awan, Wajid Aziz, Sharjil Saeed, Amjad Ali, Farukh Zeeshan, Kyung Sup Kwak

**Affiliations:** ^1^Department of Computer Science & IT, The University of Azad Jammu and Kashmir, City Campus, 13100 Muzaffarabad, Azad Kashmir, Pakistan; ^2^College of Computer Sciences and Engineering, University of Jeddah, Jeddah 21959, Saudi Arabia; ^3^Department of Computer Science, COMSATS University Islamabad, Lahore Campus, Lahore, Pakistan; ^4^Department of Information and Communication Engineering, Inha University, Incheon, Republic of Korea

## Abstract

The adaptability of heart to external and internal stimuli is reflected by the heart rate variability (HRV). Reduced HRV can be a predictor of negative cardiovascular outcomes. Based on the nonlinear, nonstationary, and highly complex dynamics of the controlling mechanism of the cardiovascular system, linear HRV measures have limited capability to accurately analyze the underlying dynamics. In this study, we propose an automated system to analyze HRV signals by extracting multimodal features to capture temporal, spectral, and complex dynamics. Robust machine learning techniques, such as support vector machine (SVM) with its kernel (linear, Gaussian, radial base function, and polynomial), decision tree (DT), k-nearest neighbor (KNN), and ensemble classifiers, were employed to evaluate the detection performance. Performance was evaluated in terms of specificity, sensitivity, positive predictive value (PPV), negative predictive value (NPV), and area under the receiver operating characteristic curve (AUC). The highest performance was obtained using SVM linear kernel (TA = 93.1%, AUC = 0.97, 95% CI [lower bound = 0.04, upper bound = 0.89]), followed by ensemble subspace discriminant (TA = 91.4%, AUC = 0.96, 95% CI [lower bound 0.07, upper bound = 0.81]) and SVM medium Gaussian kernel (TA = 90.5%, AUC = 0.95, 95% CI [lower bound = 0.07, upper bound = 0.86]). The results reveal that the proposed approach can provide an effective and computationally efficient tool for automatic detection of congestive heart failure patients.

## 1. Introduction

Heart rate variability (HRV) signals are extracted from electrocardiogram (ECG) [1], which is a noninvasive marker for monitoring an individual's health. The time interval between two consecutive R-peaks in an ECG is called an RR interval or interbeat interval. The analysis of variations in the interbeat intervals is called HRV analysis, which has diverse applications in various fields of clinical research to examine a wide range of cardiac and noncardiac diseases, including myocardial infarction (MI) [2], hypertension [3], sudden cardiac death (SCD) and ventricular arrhythmias [4], and diabetes mellitus (DM) [5]. A low or depressed HRV is seen in congestive heart failure (CHF) patients. It is hard to visually identify the minute variations in HRV signals because ECG signals contain noise and baseline shift. Thus, analysis of such type of signals using traditional methods and visual detection is challenging, inappropriate, and time-consuming. Moreover, the parameters of HRV are affected by respiration [6], instantaneous variation [7], and motion artifacts [8]. Thus, to minimize these obstacles of visual and manual interpretation, researchers developed computer-aided diagnostic (CAD) techniques for HRV analysis.

About 26 million people are suffering from CHF around the world [9]. This is the pathophysiological condition in which the heart cannot provide enough blood to meet the body's requirements [9], resulting in the reduction in the ventricle's ability to pump blood [10]. The most common indications of CHF include dyspnea, edema, fatigue [9, 10], heart valve disease, myocardial infarction (MI), and dilated cardiomyopathy [11]. CHF patients are more susceptible to sudden cardiac death [12]. Hence, CHF must be detected at the early stages. In this work, we aim to develop a system that can automatically distinguish between normal persons and CHF patients using heart rate variability (HRV) signals.

Interbeat intervals cannot be easily analyzed using visual detection, which may lead toward inaccurate classification of normal and diseased subjects. In this regard, various techniques [1] have been developed for automated detection and prediction of normal and abnormal HRV signals, including discrete wavelet transform (DWT) and empirical mode decomposition (EMD). HRV signals have been used to diagnose coronary artery disease (CAD) automatically [13]. Likewise, these signals have also been used to detect arrhythmia [14], risk of cardiovascular diseases [15], post-myocardial infarction (MI) patients, hypertension [16], diabetes [17], and sudden cardiac death [4].

Researchers [18] used time domain analysis techniques to analyze HRV signals and observed that CHF has an association with autonomic dysfunction. Frequency domain measures such as low frequency (LF), very low frequency (VLF), high frequency (HF), ratio of LF and HF, and total power from the HRV signals have been used for assessing cardiac autonomic control [17]. It was observed that VLF power is an independent risk predictor in CHF patients. A decrease in HRV has been observed in CHF patients in comparison to healthy persons [19]. Likewise, researchers [20] computed the standard deviation of normal to normal beat interval (SDNN) and used it for discriminating normal and CHF subjects. The researchers [21] analyzed the HRV signal of low-risk patients (LRP) and high-risk patients (HRPs) of CHF using time and frequency domain measures. It was observed that frequency domain parameters calculated from HRV signals were low in HRPs, except LF/HF ratio. Moreover, researchers [21] studied the dynamics of HRV in CHF patients and found lower values of standard HRV measures, except HF power. The lower values of HRV parameters have a correlation with the functional severity of heart failure [21]. Kumar et al. [22] proposed an automated method to diagnose CHF using HRV signals. This method is based on FAWT by decomposing the HRV signals into different sub-band signals. Further, accumulated permutation entropy (APEnt) and fuzzy entropy (AFEnt) are computed over cumulative sums of these sub-band signals. Soni et al. [23] proposed data mining techniques for predicting heart diseases. They observed that data mining techniques such as decision tree (DT) and Bayesian network (BN) approach outperformed other predictive methods such as KNN and neural networks. The classification accuracy of DT and BN after applying the genetic algorithm by reducing the data dimension to obtain an optimal subset of attributes improved heart disease prediction [24]. Heart rate signals are nonlinear, nonstationary, complex, and time variant. Based on these characteristics, we extracted multimodal features from these signals and used robust machine learning to distinguish NSR and CHF subjects. We used jack-knife 10-fold cross-validation and evaluated the performance in terms of sensitivity, specificity, positive predictive value, negative predictive value, and total accuracy.

## 2. Material and Methods

**Figure 1:**
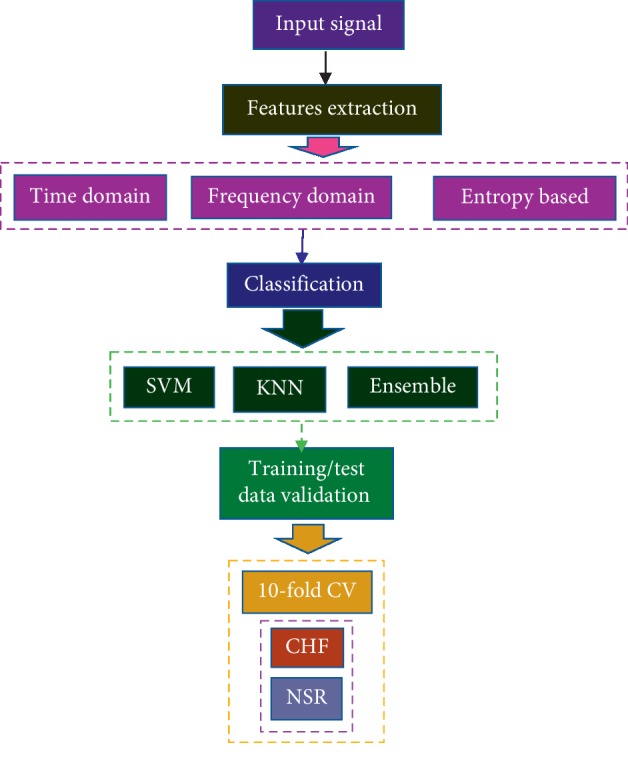
Schematic diagram for the classification of NSR and CHF subjects.

Figure 1 shows a schematic diagram to illustrate the procedure used for the classification of NSR and CHF subjects by using multimodal features.

### 2.1. Dataset

The RR interval time series data were taken from the Physionet databases [25]. The fluctuations in the cardiac interbeat interval (RR interval) time series data of normal sinus rhythm (NSR) subjects, congestive heart failure (CHF) subjects, and atrial fibrillation (AF) subjects were studied [25]. The data of NSR subjects were taken from 24-hour Holter monitor recordings of 72 subjects consisting of 35 men and 37 women (54 from the RR interval normal sinus rhythm database and 18 from the MIT-BIH normal sinus rhythm database). The age of the measured group was 54.6 ± 16.2 years (mean ± SD), range 20–78 years. ECG data were sampled at 128 Hz. The CHF group comprised 44 subjects, 29 men and 15 women aged 55.5 ± 11.4 years, range 22–78 years. The data of 29 CHF subjects were obtained from the RR interval congestive heart failure data and 15 from the MIT_BIH Bidmic congestive heart failure database [25]. CHF subjects can be classified into four groups according to the New York Heart Association (NYHA) functional classification system. This system classifies patients according to the symptoms to everyday activity and quality of life of patients. In this study, we considered 20,000 samples for all subjects, including both CHF and NSR subjects, while extracting features.

### 2.2. Feature Extraction

In most of the classification and regression problems, the first and foremost step is to extract the most relevant features. To predict colon cancer, researchers in the past [26] extracted hybrid and geometric features. Moreover, to detect breast cancer, Dheeba et al. [27] extracted texture features. Wang et al. [28] extracted multimodal features from multimodal domains such as time domain, frequency domain, and complexity-based features to detect epileptic seizure. This will give a unified framework to include the advantages of varying characteristics of EEG signals. Moreover, nonlinear dynamics based on the KD tree algorithm (fast sample entropy) provide better results than the traditional entropy methods.

To capture the temporal short-, medium-, and long-term dynamics from the physiological signals and systems, we computed the time domain features from the CHF and normal subjects. Moreover, for spectral dynamics, we extracted the frequency domain features. The statistical features were also computed to capture basic statistical properties from these signals. Moreover, most of the physiological signals are nonlinear in nature and contain complex hidden dynamics, which can be best detected using entropy-based computational features. Thus, in this study, we extracted linear features such as time domain, frequency domain, and statistical and nonlinear features, such as entropy-based complexity features and wavelet entropy features, to differentiate normal subjects from CHF subjects. In order to judge the efficiency of the features, we applied *t*-test and ROC curve as previously employed by the researchers using different rank tests [29–31].

#### 2.2.1. B.B.A. Linear Methods

To measure the variability in physiological signals (i.e., EEG or ECG) affected by different pathologies, the time and frequency domain methods are widely used to capture the time and spectral dynamics in these signals. The time domain methods are used to capture the short-, medium-, and long-term variations present in the physiological signals and systems, whereas to capture the dynamics present in different spectra, frequency domain features are computed. There are literature evidences [32, 33] for patients who suffered from different variability dysfunctions [34–40], including heart rate variability, breathing, depression, pulse variability, insomnia problems, and epilepsy.

#### 2.2.2. B.B.B. Nonlinear Methods

Biological signals are the output of multiple interacting components and exhibit complicated patterns and rhythms. These rhythmical changes and patterns contain very useful hidden information to study the underlying dynamics of these systems. It is unrealistic to extract valuable information using traditional data analysis techniques. The complexity of the physiological systems comprised structural components and coupling among them, which is degraded with aging and disease. Following are the most commonly used complexity base measures as detailed in [28]. The complexity of healthy subjects computed using entropy methods is higher than that of diseased subjects. The reason behind this analogy is that all the structural components and coupling functions among the structural components in these healthy subjects are properly working and connected for communication, thereby increasing their entropy values and complexity. On the other hand, the entropy and complexity of the diseased subjects are reduced because of the degradation of the coupling among the structural components.

#### 2.2.3. Approximate Entropy

Pincus in 1991 proposed approximate entropy (ApEn) [41] to quantify the regularity present in the time series data. This entropy measure indicates that the probability of similar observation patterns does not repeat. Mathematically,

(1) $$\begin{array}{c} \text{ApEn}\left(m,r,\;N\right)=\emptyset^{m}\left(r\right)-\emptyset^{m+1}\left(r\right). \end{array}$$

To compute the approximate entropy, two criteria are set, i.e., *m*, which is the window length and *r*, the similarity criteria. In this study, we choose *m* = 3 and *r* = 0.15 times the standard deviation of data as offered in [41].

#### 2.2.4. Fast Sample Entropy with KD Tree Approach

Sample entropy (SampEn) as proposed by [42] is a modified form of approximate entropy. Sample entropy in comparison to approximate entropy is more robust because it is independent of data length and trouble-free implementation.

Bentley in 1975 developed a binary tree algorithm known as Kd tree algorithm. Its each “v” node is associated with a rectangle Bv. If Bv does not contain any point in its interior, the “v” will be the leaf node. In other cases, Bv will be partitioned into two rectangles by drawing a horizontal and a vertical line such that each rectangle contains at most half of the points. The computation of Kd tree algorithm is detailed by [28]. The time and space complexity is reduced using the following steps.

  Step 1. Transform the original discrete time series to a special set from *x*={*x*1, *x*2, *x*3,…, *xN*}.
  Step 2. The d-dimensional kd tree is constructed using *N*-m points for which the total cost is *O*(*N* long *N*) and memory is *O*(*N*).
  Step 3. Range query; For d-dimensional kd search, the time cost is *NO*(*N*^1(1/*d*)^) for *N* queries and the memory cost is *O*(*N*).

#### 2.2.5. Wavelet Entropy

Researchers in the past also computed wavelet-based entropic measures to capture the nonlinearity present in the data. The most common wavelet entropy methods [43] include Shannon, Threshold, Log Energy, Sure, and Norm. Shannon entropy [43] was employed to measure signal complexity by computing wavelet coefficients generated from wavelet packets (WPT), where larger values show high uncertainty process and, therefore, higher complexity. Moreover, Rosso et al. [44] employed wavelet entropy to capture the underlying dynamical process associated with the signal. The entropy “*E*” must be an additive information cost function such that *E*(0) = 0.

(2) $$\begin{array}{c} E\left(S\right)=\;\sum_{i}E\left(S_{i}\right), \end{array}$$

where on an orthonormal basis *S* is the signal and (*S*_*i*_) are the signal coefficients, and *P* is the threshold, which is always greater than or equal to 0.

The Wentropy method was used to compute the wavelet entropy as shown below.

**Figure 2:**
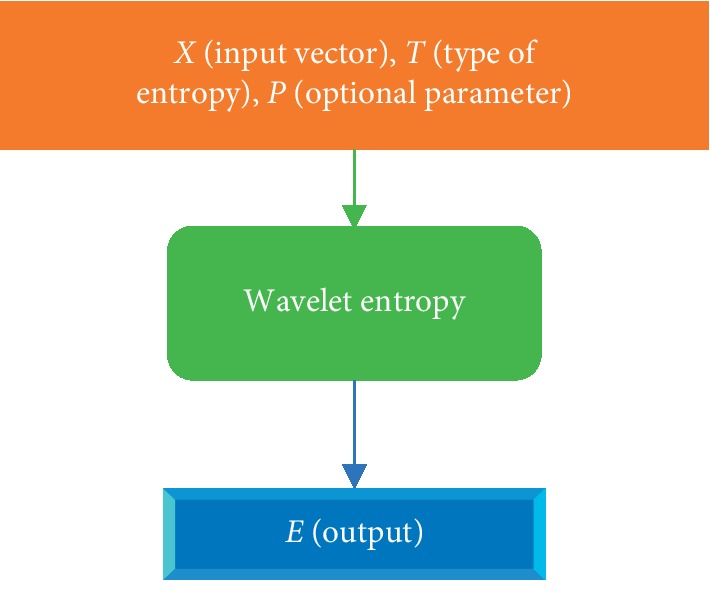
Computation of wavelet entropy.

Figure 2 depicts the flow of computing wavelet entropy by selecting different wavelet functions, such as threshold, norm, sure, and log energy.

The computation of wavelet entropy packets (Shannon, norm, log energy, threshold, and sure) as reflected in equations (3)–(9) is detailed in [45–47].

#### 2.2.6. Shannon Entropy

In 1948, Claude Shannon first proposed the Shannon entropy [48], which is most widely used in the information sciences. Moreover, it is a measure of the uncertainty linked with a random variable. Specifically, Shannon entropy quantifies the expected value of the information contained in a message. The Shannon entropy of a random variable *X* can be defined as follows:

(3) $$\begin{array}{c} V\left(X\right)=V\left(P_{1},\ldots ,P_{n}\right)=\;-\sum_{i=1}^{n}P_{i}\log_{2}P_{i}, \end{array}$$

(4) $$\begin{array}{c} P_{i}=P_{r}\;\left(X=x_{i}\right), \end{array}$$

where *P*_*i*_ is defined in equation (3), with *x*_*i*_ indicating the *i*th possible value of *X* out of *n* symbols, and *P*_*i*_ denoting the possibility of *X*=*x*_*i*_.

#### 2.2.7. Wavelet Norm Entropy

This entropy measure proposed by [49] can be mathematically expressed as follows:

(5) $$\begin{array}{c} E\left(S\right)=\frac{\sum_{i}{\left|S_{i}\right|}^{p}}{N}, \end{array}$$

where *p* is the power and must be 1 ≪ *P* < 2 the terminal node signal and (*si*) is the waveform of terminal node signals.

#### 2.2.8. Threshold Entropy

The threshold entropy was computed with threshold at 0.2.

#### 2.2.9. Sure Entropy

The parameter *P* is used as threshold and the values of *P* ≥ 0.

(6) $$\begin{array}{c} E\left(s\right)=n-\#\left\{i\text{ such that }\left|si\right|\leq p\right\}+\sum_{i}\min_{\left(si^{2},p^{2}\right)}, \end{array}$$

(7) $$\begin{array}{c} H_{\text{Sure}}\left(B\right)=-\sum_{i=1}^{N-1}P_{i}\left(B\right)\log\;2\left(P_{i}\left(B\right)\right). \end{array}$$

The Sure entropy was computed with threshold at 3.

#### 2.2.10. Norm Entropy

In norm entropy, *p* is used as power and the value of *P* ≥ 1. The concentration in l^*p*^ norm entropy is as follows:

(8) $$\begin{array}{c} E\left(si\right)={\left|s_{i}\right|}^{p},\\ \text{so }E\left(s\right)=\sum_{i}{{\left|s_{i}\right|}^{p}}_{i}={\left\|S\right\|}_{p}^{p}. \end{array}$$

The norm entropy was computed with power at 1.1.

#### 2.2.11. Log Energy

(9) $$\begin{array}{c} H_{\log\;En}\left(B\right)=-\sum_{i=0}^{N-1}{\left(\log_{2}\left(\;P_{i}\left(B\right)\right)\right)}^{2}, \end{array}$$

where *P*_*i*_(*B*) denotes the probability distribution function and is a logarithmic sum of the square of these probabilities' distribution.

## 3. Classification

Classification is a process of categorizing based on the extracted features. In machine learning, there are different types of classification techniques, such as supervised, unsupervised, and re-enforced learning. Researchers in the past employed robust machine learning classifiers such as support vector machine (SVM), decision tree (DT), K-nearest neighbors (KNNs), and Naïve Bayes, and ensemble classifiers in detecting and predicting colon cancer [26, 50]. Thus, in this study, we employed supervised learning based on label class data including support vector machine (SVM), decision tree (DT), K-nearest neighbor (KNN), and ensemble classifiers.

### 3.1. Support Vector Machine

Support vector machine (SVM) is the most important technique of supervised learning methods, which is also used for classification purposes. For solving the problems related to pattern recognition [51], medical analysis area [52, 53] and machine learning [54], recently SVM, are used. Furthermore, SVMs are also used in many other fields, such as detection and recognition, recognition of text, image retrial based on contents, biometric systems, and speech recognition. To build a single hyperplane or a set of hyperplanes in infinite space or high dimension, SVM is used. For obtaining a good classification, this hyperplane may also be used. By implementing this, a hyperplane that has the greatest distance to the nearby training point of any class is achieved. Usually, a lower generalization fault of the classifier is achieved by a larger margin.

Support vector machine tries to find a hyperplane that gives the training example with the greatest minimum distance. In support vector machine theory, this is also termed as margin. For maximized hyperplanes, the best margin is attained. There are additional significant characteristics for SVM that provide better generalization results. Support vector machine mainly has a two-type classifier which converts data into a hyperplane dependent on data that are nonlinear or dimensionally higher. The SVM hyperplane, maximizing margin, and the kernel tricks as reflected in equations (10)–(17) are detailed in [55–57].

Let us express a hyperplane by: *x* · *w*+*b*=0. Here, *w* is a normal. The data that are separated linearly are labeled as follows:

(10) $$\begin{array}{c} \left\{x_{i},y_{i}\right\},x_{i}\in R^{N}d,y_{i}\in \left\{-1,1\right\},\;i=1,2,\ldots ,N, \end{array}$$

where *y*_*i*_ is used as a two-class SVM class label. When objective function is maximized, the boundary obtained is optimum with the greatest margin: *E*=*w*^2^ gives

(11) $$\begin{array}{c} \begin{array}{cc} x_{i}\cdot w+b\geq 1, & \text{for }y_{i}=+1,\\ x_{i}\cdot w+b\leq 1, & \text{for }y_{i}=-1. \end{array} \end{array}$$

Combining these into a set of dissimilarities as

(12) $$\begin{array}{c} \left(x_{i}.b+b\right)y_{i}\geq 1,\;\text{for all }i. \end{array}$$

**Figure 3:**
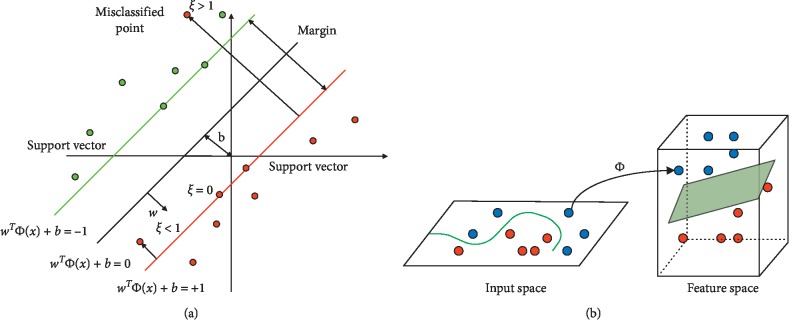
(a) Error on margin using slack variable, (b) SVM nonlinear separation.

When the data are not linearly separable, then a slack variable *Ξ*_*i*_ to represent the amount of misclassification rate is used as reflected in Figures 3(a) and 3(b). Thus, the objective function in this case can be defined as

(13) $$\begin{array}{c} E=\frac{1}{2}{\left\|w\right\|}^{2}+C\sum_{i}L\left(\Xi_{i}\right). \end{array}$$

Subject to

(14) $$\begin{array}{c} \left(x_{i}.b+b\right)y_{i}\geq 1-\xi_{i},\;\text{for all }i. \end{array}$$

On the right-hand side, the first term denotes the regularization term which gives the ability to SVM for generalization on the sparse data, whereas the second term represents the empirical risk for the points that lie within the margin or are misclassified. Here, *L* represents the cost function and C denotes the hyper-parameter, which shows the trade-off effect by minimising the empirical risk against maximizing the margin. To detect the outlier, the linear error cost function is used. The dual formulation with *L*(*Ξ*_*i*_)=*Ξ*_*i*_ is

(15) $$\begin{array}{c} \alpha^{*}=\max_{\alpha }\left(\underset{i}{\sum }\alpha_{i}+\underset{i,j}{\sum }\alpha_{i}\;\alpha_{j}\;y_{i}\;y_{j}\;x_{i}\;x_{j}\right). \end{array}$$

Subject to

(16) $$\begin{array}{c} 0\leq \alpha_{i}\leq C,\\ \underset{i}{\sum }\alpha_{i}\;y_{j}=0. \end{array}$$

Here, *α*={*α*_1_, *α*_2_, *α*_3_,…, *α*_*i*_} is a set of Lagrange multipliers of the constraints in the primal optimization problem. The optimal decision boundary is now given by

(17) $$\begin{array}{c} w_{0}=\sum_{i}\alpha_{i}x_{i}y_{i}. \end{array}$$

#### 3.1.1. Kernel Trick

For data that are not linearly separable, Muller et al. [59] recommended kernel trick to handle this type of data. To cope up with this type of problem, the nonlinear mapping function from the input space is transformed into a higher dimensional feature space. Thus, in the input space, the dot product between two vectors is expressed by the dot product with some kernel functions in the feature space. The commonly used kernel functions are as follows.

#### 3.1.2. SVM Polynomial Kernel

(18) $$\begin{array}{c} K\left(x_{i},y_{i}\right)={\left(x_{i}.y_{i}+1\right)}^{n}. \end{array}$$

#### 3.1.3. SVM Gaussian (RBF) Kernel

(19) $$\begin{array}{c} K\left(x_{i},y_{i}\right)=\exp\left(\frac{-1}{2}\frac{{\left\|x_{i}-y_{i}\right\|}^{2}}{\sigma^{2}}\right). \end{array}$$

#### 3.1.4. SVM Fine Gaussian (RBF) Kernel

(20) $$\begin{array}{c} K\left(x_{i},y_{i}\right)=\exp\left(\frac{-1}{2}\frac{{\left\|x_{i}-y_{i}\right\|}^{\prime }\left\|x_{i}-y_{i}\right\|}{\sigma^{2}}\right), \end{array}$$

where *n* is the order of polynomial kernel and *σ* is the width of RBF. The dual formulation for a nonlinear case is given by

(21) $$\begin{array}{c} \alpha^{*}=\max_{\alpha }\left(\sum_{i}\alpha_{i}+\sum_{i,j}\alpha_{i}\;\alpha_{j}\;y_{i}\;y_{j}K\left(x_{i}\cdot x_{j}\right)\right). \end{array}$$

Subject to

(22) $$\begin{array}{c} 0\leq \alpha_{i}\leq C,\\ \sum_{i}\alpha_{i}\;y_{j}=0. \end{array}$$

The performance of SVM classifiers depends on several parameters. One of the famous methods is the grid search method, which selects the optimal parameter value by setting carefully the grid range and step size. In linear kernel, only one parameter, i.e., “*c*” a soft margin constant, is used, which represents the constraint violation cost associated with the data point lying on the wrong side of the decision boundary. However, the SVM with Gaussian and RBF has two training parameters: cost (*c*), which controls the overfitting of the model, and sigma (), which controls the degree of nonlinearity of the model. In this study, we used the default values of both cost function and sigma. For SVM fine Gaussian, the default kernel scale was selected as 0.61; for medium Gaussian, the kernel scale was 2.4; and for coarse Gaussian, the kernel scale was 9.8.

### 3.2. Decision Tree (DT)

The DT classifier checks the dataset similarity that is given and classifies it into different separate classes. Decision trees are used for making classifiers of data depending on the choice of a feature, which fixes and maximizes the data division. These attributes are separated into different branches until the end criteria are met. The mathematical formulations are described in [59] for equations (23) and (24).

**Figure 4:**
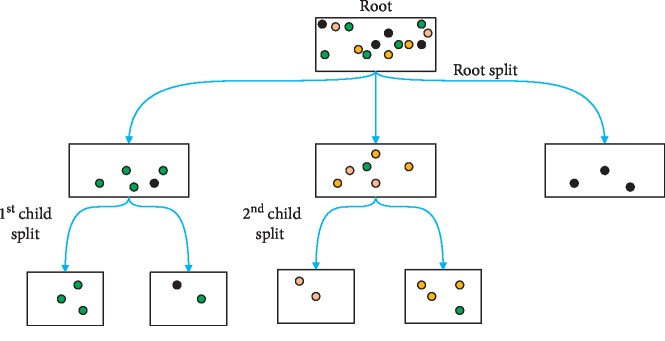
Decision tree split decision.

The decision tree classifier is based on supervised learning technique, which uses a recursive approach by dividing a dataset in order to reach a similar classification of a goal like below (Figure 4.

Mathematically, the following algorithm is used to compute the DT:

(23) $$\begin{array}{c} \bar{X\;}=\;{\left\{X_{1},X_{2},X_{3},\ldots ,X_{m}\right\}}^{T},\\ X_{i}=\left\{x_{1},x_{2},x_{3},\ldots ,x_{ij},\ldots ,x_{in}\right\},\\ S=\left\{S_{1},S_{2},S_{3},\ldots ,S_{i},\ldots ,S_{m}\right\}. \end{array}$$

In the above equations, *m* denotes the available quantity of observations, *n* denotes the number of independent variables, *S* denotes the m-dimension vector of the variable predicted through $\bar{X}\;\cdot X_{i}$ is the *i*^th^ element of *n*-dimension independent variables. The independent variable is *x*_1_, *x*_2_, *x*_3_,…, *x*_*ij*_,…, *x*_*in*_ of design *X*_*i*_ vector and *T* is used for transpose symbolization.

The main aim of DT is to estimate the value of $\bar{X}$. By using $\bar{X}$, different DTs may construct different accuracy and correctness levels; however, an optimum DT is inspiring because the space for search has a larger dimension.

To find the trade-off between correctness and complication for decision trees, appropriate algorithms can be created. In this situation, a categorization of locally optimum decisions that are nearly the parameters of features is used for making partition of the dataset $\bar{X}$ using algorithms of DTs. Optimum DT, *T*_*k*0_, is created according to the following problems of optimization.

(24) $$\begin{array}{c} \hat{R}\left(T_{k0}\right)=\min\left\{\hat{R}\;\left(T_{k0}\right)\right\},\;k=1,2,3,\ldots ,K,\\ \hat{R}\left(T\right)=\sum_{t\in T}^{k}\left\{r\left(t\right)p\left(t\right)\right\}, \end{array}$$

where $\hat{R}\;\left(T\right)$ symbolizes the level of error during the misclassification of tree *T*_*k*_, *T*_*k*0_ indicates the optimum decision tree that reduces the error related to misclassification in the binary tree, and *T* denotes the binary tree ∈, {*T*_1_, *T*_2_, *T*_3_,…, *T*_*k*_, *t*_1_}. The tree index is represented by *k*, *t* stands for node of tree, *t*_1 _ stands for node of root, *r*(*t*) for resubstituting error that misclassifies node *t*, *p*(*t*) represents the probability that any case drop into node *t* is represented by *T*^*L*^ and *T*^*R*^ representing the sub-trees of the right and left sets of partition. The tree *T* is deliberate by feature plan partitioning.

Most of the classification problems with large datasets are complex and contain errors, and the decision tree algorithm is most appropriate in these situations. The decision tree works by taking the objects as an input and giving the output as yes/no decision. Decision trees use sample selection [60] and also exhibit Boolean functions [61]. The decision trees are also quick and effective methods used for large classification dataset entries and provide best decision support proficiencies. There are many applications of using DTs, such as medical problems, and economic and other scientific situations [62].

There are several parameters that are used to tune the decision tree. In this study, we used the default parameters to get a baseline. The min-sample-per-leaf node was set to 1 by default, which can make a tree over fit and learn from all the data points, including outliners. Another parameter is the maximum depth of the tree, which indicates how deep the tree can be. A deeper tree has more splits and is capable of capturing more information about the data. The decision tree in this study was fit with a depth ranging from 1 to 32. Another important parameter is the number of random splits required to split the internal node. This varies from considering at least one sample at each node by considering all samples at each node. By increasing the parameters, the tree can become more constrained because it will consider more samples at each node. In this study, we consider this parameter from 10% to 100% of the sample. A similar approach was adopted for minimum sample leaf.

#### 3.2.1. K-Nearest Neighbor (KNN)

In the field of pattern recognition, machine learning, etc., K-nearest neighbor is the regularly used algorithm. KNN is a nonparametric method used for both classification and regression problems. In both cases, the given input consists of k-closest training samples in the feature space. The output is dependent on whether we use KNN for regression or classification. For the KNN classification method, the output is a class membership. Any object can be classified based on the majority voting of its neighboring data points with the object being assigned to the class that is common among its K-nearest neighbors (where K is a positive integer, typically small). If K = 1, then the objects will be classified and assigned to the nearest class of that single neighbor.

We used the default parameters during training/testing of data using the KNN algorithm. KNN was used for classification complications in [63]. KNN is also termed as lazy learning algorithm. A classifier is not promptly constructed; however, all preparation information tests are spared and held up until the point that new perceptions should be classified. These characteristics of the lazy learning algorithm make it better than excited learning because it builds a classifier even before new interpretations need to be classified. It is explored by [64] that KNN is also more important when the dynamical data need to be changed and more rapidly simplified. Different distance matrices are employed for KNN. The following are steps of this algorithm in which the formula of Euclidean distance are used and reflected by equation (25) (also described in [65]).

**Step 1 I.**

In the first step, prepare the framework and provide the feature space to KNN.

**Step 2 II.**

By using the following distance formula termed as Euclidean distance formula, find the distance.

(25) $$\begin{array}{c} d\left(x_{i},y_{i}\right)=\sum_{i=1}^{n}\sqrt{{\left(x_{i}-y_{i}\right)}^{2}}. \end{array}$$

**Step 3 III.**

Type the calculated value from the Euclidean distance formula by using *d*_*i*_ ≤ *d*_*i*_+1, where *i*=1,2,3,…, *k*.

**Step 4 IV.**

According to the nature of data, apply different means and polling.

**Step 5 V.**

The value of K (i.e., the number of nearest neighbors) depends on the volume and nature of data delivered to KNN. For smaller data, the value of *k* is also reserved small, and for large data, the value of *k* is reserved as large.

In this study, we selected *K* = 3, distance metrics as Euclidean distance, and distance weight as equal weight.

#### 3.2.2. Ensemble Classifiers

The ensemble classifiers comprise a set of individually trained classifiers whose predictions are then combined when classifying the novel instances using different approaches. These new learning algorithms by constructing a set of algorithms classify new data based on the new data points by taking the weight of their prediction. Based on these capabilities, these algorithms have successfully been used to enhance the prediction power in a variety of applications, such as predicting signal peptide for predicting protein subcellular location [66], predicting subcellular location, and predicting enzyme subfamily prediction [67]. The ensemble classifiers in many applications give relatively enhanced performance than the individual classifier. The researchers [68] reported that individual classifiers can produce different errors during classification; however, these errors can be minimized by combining the classifiers because the error produced by one classifier can be compensated by another classifier.

#### 3.2.3. Performance Evaluation Measures

To detect CHF, the following measures were used to compute the true positive rate (TPR), true negative rate (TNR), positive predictive value (PPV), negative predictive value (NPV), total accuracy (TA), and area under the receiver operating curve (AUC) as depicted in equations (21)–(25) and detailed in [69, 70].

#### 3.2.4. True Positive Rate (TPR)

The TPR measure, also known as sensitivity or recall, is used to test the proportion of people who test positive for the disease among those who have the disease. Mathematically, it is expressed as follows:

(26) $$\begin{array}{c} \text{TPR}=\frac{\sum \text{True Positive}}{\sum \text{Condition Positive}},\\ \text{TPR}=\frac{\text{TP}}{\text{TP}+\text{FN}}, \end{array}$$

i.e., the probability of positive test given that the patient has the disease.

#### 3.2.5. True Negative Rate (TNR)

The TNR measure also known as Specificity is the proportion of negatives that are correctly identified. Mathematically, it is expressed as

(27) $$\begin{array}{c} \text{TNR}=\frac{\sum \text{True Negative}}{\sum \text{Condition Negative}},\\ \text{TNR}=\frac{\text{TN}}{\text{TN}+\text{FP}}, \end{array}$$

i.e. probability of a negative test given that patient is well.

#### 3.2.6. Positive Predictive Value (PPV)

PPV is mathematically expressed as follows:

(28) $$\begin{array}{c} \text{PPV}=\frac{\sum \text{True Positive}}{\sum \text{Predicted Condition Positive}},\\ \text{PPV}=\frac{\text{TP}}{\text{TP}+\text{FP}}, \end{array}$$

where TP denotes that the test makes a positive prediction and the subject has a positive result under gold standard, while FP is the event that the test makes a positive prediction and the subject has a negative result.

#### 3.2.7. Negative Predictive Value (NPV)

NPV can be computed as

(29) $$\begin{array}{c} \text{NPV}=\frac{\sum \text{True Negative}}{\sum \text{Predicted Condition Negative}},\\ \text{NPV}=\frac{\text{TN}}{\text{TN}+\text{FN}}, \end{array}$$

where TN indicates that the test makes a negative prediction and the subject also has a negative result, while FN indicates that the test makes a negative prediction and the subject has a positive result.

#### 3.2.8. Total Accuracy (TA)

The total accuracy is computed as

(30) $$\begin{array}{c} \text{TA}=\frac{\text{TP}+\text{TN}}{\text{TP}+\text{FP}+\text{FN}+\text{TN}}. \end{array}$$

#### 3.2.9. The 95% Confidence Interval (CI)

For the mean *μ*_*X*_, a common confidence interval is 95% CI. For normally distributed sample means, *z*-statistics (called *z*1 and *z*2) is such that P (*z*1 < *Z* < *z*2) = 0.95.

The margin of error can be computed by multiplying the value of *Z*2, denoted by *Z*^*∗*^, by the standard deviation of the sample mean, i.e., $\delta_{\bar{X}}=\delta_{X}/\sqrt{n}$. That is, the margin of error is $\left(Z^{*}\right)\left(\delta_{X}/\sqrt{n}\right)$.

The lower bound and upper bound as reflected in equations (31) and (32) are detailed in [71, 72].

#### 3.2.10. Lower Bound (LB) of 95% CI

The lower bound of 95% CI for *μ*_*X*_ is computed by subtracting the margin of error from the point estimate $\bar{X}$:

(31) $$\begin{array}{c} \text{lower bound }\left(\text{LB}\right)=\;\bar{X}-\;\left(Z^{*}\right)\left(\frac{\delta_{X}}{\sqrt{n}}\right). \end{array}$$

#### 3.2.11. Upper Bound (UB) of 95% CI

The upper bound of 95% CI for *μ*_*X*_ is computed by adding the margin of error with the point estimate $\bar{X}$:

(32) $$\begin{array}{c} \text{upper bound }\left(\text{UB}\right)=\bar{X}+\left(Z^{*}\right)\left(\frac{\delta_{X}}{\sqrt{n}}\right). \end{array}$$

## 4. Results

**Figure 5:**
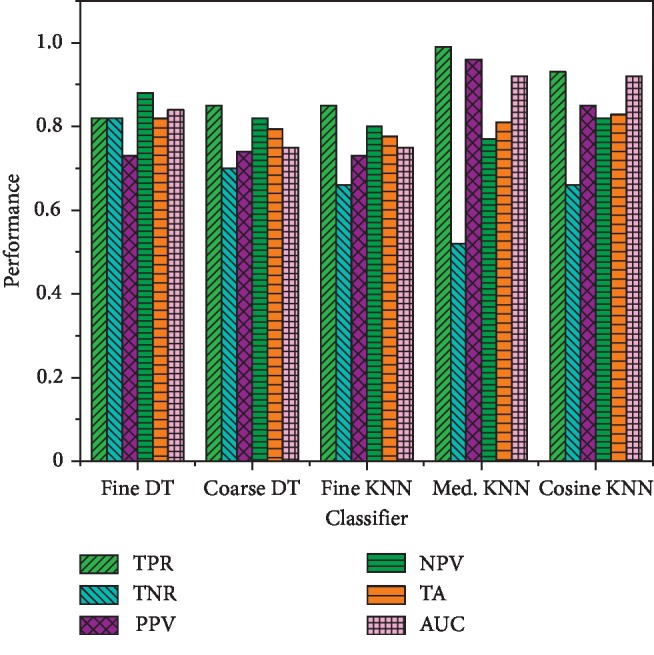
Heart failure rate detection performance using decision tree and KNN methods.

**Figure 6:**
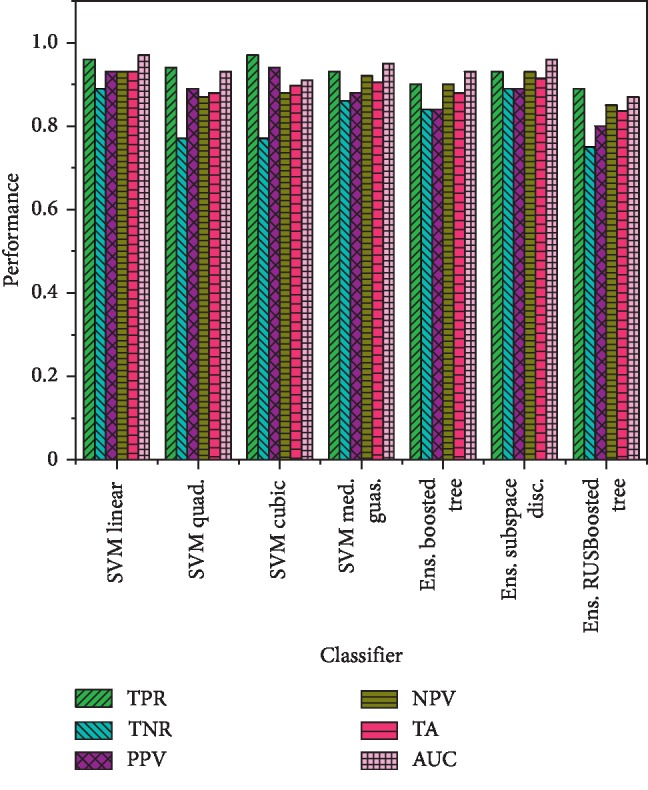
Heart failure rate detection performance using SVM and ensemble methods.

**Figure 7:**
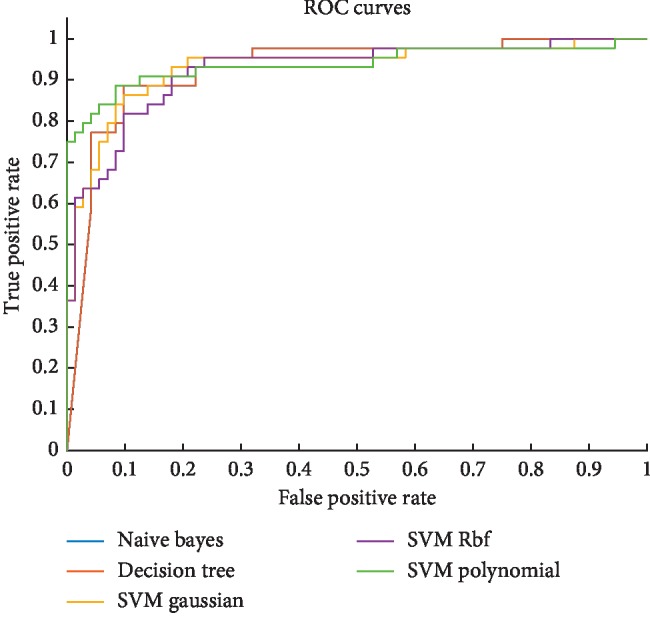
ROC to detect heart failure using Naïve Bayes, decision tree, and SVM with its kernels.

In this study, we extracted multimodal features, such as time domain, frequency domain, statistical and complexity-based features from congestive heart failure (CHF), and normal sinus rhythm (NSR) subjects. We computed the performance based on single features and hybrid features. Robust machine learning classification methods, such as decision tree (DT), support vector machine (SVM) and its kernel, K-nearest neighbors (KNN), and ensemble methods, were employed. The performance was computed using true positive rate (TPR), true negative rate (TNR), positive predictive value (PPV), negative predictive value (NPV), total accuracy (TA), and area under the receiver operating curve (AUC). Performance based on single features is reflected in Tables 1–4, whereas performance based on a combination of features is reflected in Figures 5–7.

We extracted the time domain features, such as SDANN, SDNN, SDSD, and RMSSD, and applied machine learning classifiers such as decision tree (DT); support vector machine (SVM) and its kernels linear, quadratic, cubic, and medium Gaussian; K-nearest neighbor (KNN) with fine, medium, and cosine KNN; and ensemble classifiers such as bagged tree, subspace discriminant, and RUSBoosted tree, as reflected in Table 1. The detection performance with decision tree such as fine DT was obtained, such as TPR (78%), TNR (77%), PPV (68%), NPV (77.8%), TA (77.6%), AUC (0.73), and 95% CI with LB (0.22) and UB (0.77). Using coarse tree, we obtained the performance such as TPR (89%), TNR (55%), PPV (78%), NPV (81%), TA (80.2%), and AUC (075) with LB (0.11) and UB (0.66). Similarly, the highest detection performance was obtained using SVM linear with TPR (90%), TNR (73%), PPV (82%), NPV (84%), TA (83.6%), and AUC (0.92) with LB (0.10) and UB (0.73), followed by SVM medium Gaussian with TPR (89%), TNR (73%), PPV (80%), NPV (80%), PPV (84%), TA (82.8%), and AUC (0.90) with LB (0.11) and UB (0.73); SVM cubic with TA (79.3%), AUC (0.88) and SVM quadratic with TA (79.3%) and AUC (0.84). Likewise, by applying KNN, the highest detection accuracy was obtained using cosine KNN with TA (81.0), AUC (0.83) followed by medium KNN with TA (80.2%), AUC (0.87) and fine KNN with TA (71.6%), AUC (0.69). By applying the ensemble classifiers, the highest detection performance was obtained using subspace discriminants with TPR (96%), TNR (66%), PPV (91%), NPV (82%), TA (84.5%), and AUC (0.91) with LB (0.04) and UB (0.66), followed by bagged tree with TA (81.0%), AUC (0.87), and RUSBoosted tree with TA (73.3%), AUC (0.81).

By extracting the frequency domain features such as TP, ULF, VLF, LF, HF, and LF/HF from CHF and normal subjects, as reflected in Table 2, we applied different machine learning classifiers to distinguish these conditions. Using the decision tree, the highest detection performance was obtained with coarse DT such as TA (81.9%), AUC (0.81) followed by fine DT with TA (80.2%), AUC (0.84). Using SVM, the highest detection accuracy was obtained using SVM medium Gaussian with TA (85.3%), AUC (0.90) followed by quadratic SVM with TA (81.9%), AUC (0.88); linear SVM with TA (80.2%), AUC (0.86); and cubic SVM with TA (%), AUC (0.83). Likewise, by applying KNN, the highest detection performance was obtained using fine KNN with TA (81.0%), AUC (0.86) followed by medium KNN with TA (80.2%), AUC (0.88) and cosine KNN with TA (67.2%), AUC (0.75). Moreover, by applying the ensemble classifiers, the highest detection performance was obtained using bagged tree with TA (81.9%), AUC (0.88) followed by subspace discriminant with TA (80.2%), AUC (0.85) and RUSBoosted tree with TA (77.6%), AUC (0.81).

To discriminate the CHF from normal subjects, we extracted statistical features such as RMS, variance, skewness, smoothness, and kurtosis, as reflected in Table 3, and applied robust machine learning techniques. Based on decision tree, the highest detection performance was obtained using coarse DT with TA (77.6%), AUC (0.80), followed by fine DT with TA (75.9%), AUC (0.77). Similarly, by applying SVM, the highest detection performance was obtained using SVM linear with TA (81.9%), AUC (0.80), followed by SVM quadratic with TA (81.0%), AUC (0.84); SVM medium Gaussian with TA (73.9%), AUC (0.81), and SVM cubic Gaussian with TA (75.9%), AUC (0.78). By applying KNN, the highest detection accuracy was obtained using cosine KNN with TA (73.3%), AUC (0.78), followed by medium KNN with TA (71.6%), AUC (0.78) and fine KNN with TA (69.0%), AUC (0.66). Likewise, by applying the ensemble classifiers, the highest detection accuracy was obtained using bagged tree with TA (77.6%), AUC (0.81), followed by RUSBoosted tree with TA (77.6%), AUC (0.79) and subspace discriminant with TA (74.1%), AUC (0.77).

The entropy-based features were computed based on complexity measures such as sample entropy using KD tree approaches; approximate entropy and wavelet entropy measures such as Shannon, threshold, log energy, sure, and norm; and applied machine learning classifiers such as DT, SVM, KNN and ensemble classifiers, as reflected in Table 4. By applying the decision tree, the highest detection performance was obtained using coarse DT with TA (69.8%), AUC (0.65), followed by fine DT with TA (62.9%), AUC (0.65). Likewise, using SVM, the highest detection accuracy was obtained using SVM quadratic with TA (73.3%), AUC (0.74), followed by SVM cubic with TA (70.7%), AUC (0.73); SVM medium Gaussian with TA (69.8%), AUC (0.75); and SVM linear with TA (69.0%), AUC (0.71). By applying KNN, the highest detection performance was obtained using medium KNN with TA (71.6%), AUC (0.69), followed by cosine KNN with TA (70.7%), AUC (0.72) and fine KNN with TA (68.1%), AUC (0.66). Similarly, by applying the ensemble classifiers, we obtained the highest detection performance using bagged tree with TA (72.4%), AUC (0.78), followed by subspace discriminant with TA (69.8%), AUC (0.71) and RUSBoosted tree with TA (69.0%), AUC (0.75).

Based on a combination of features, the detection performance using DT and KNN is shown in Figure 5. The performance obtained using decision tree (DT) with fine DT was obtained as TPR (82%), TNR (82%), PPV (73%), NPV (88%), TA (81.9%), and AUC (0.84) and with coarse DT as TPR (85%), TNR (70%), PPV (74%), NPV (82%), TA (79.3%), and AUC (0.75). The performance based on KNN was obtained as fine KNN with TPR (85%), TNR (66%), PPV (73%), NPV (80%), TA (77.6%), and AUC (0.75); median KNN with TPR (99%), TNR (52%), PPV (96%), NPV (77%), TA (81%), and AUC (0.92); and cosine KNN with TPR (93%), TNR (66%), PPV (85%), NPV (82%), TA (82.8%), and AUC (0.92).

The heart failure rate detection performance based on SVM was obtained using SVM linear as TPR (96%), TNR (89%), PPV (93%), NPV (93%), TA (93.1%), and AUC (0.97); SVM quadratic with TPR (94%), TNR (77%), PPV (89%), NPV (87%), TA (87.9%), and AUC (0.93); SVM cubic with TPR (97%), TNR (77%), PPV (94%), NPV (88%), TA (89.7%), and AUC (0.91); and SVM median Gaussian with TPR (93%), TNR (86%), PPV (88%), NPV (92%), TA (90.5%), and AUC (0.95). The performance computed using ensemble methods was obtained using ensemble boosted tree with TPR (90%), TNR (84%), PPV (84%), NPV (90%), TA (87.9%), and AUC (0.93); ensemble subspace discriminant with TPR (93%), TNR (89%), PPV (89%), NPV (93%), TA (91.4%), and AUC (0.96); and ensemble RUSBoosted tree with TPR (89%), TNR (75%), PPV (80%), NPV (85%), TA (83.6%), and AUC (0.87). The detection performance is shown in Figure 6.

Figure 7 depicts the heart failure rate detection performance using area under the receiving operating curve (ROC). Multimodal features based on entropy methods, wavelets, statistical, time, and frequency domain features are extracted from congestive heart failure and normal subjects. Based on the combined features, the highest AUC was obtained using SVM RBF with AUC (0.9359), followed by SVM Gaussian with AUC (0.9293), Naïve Bayes and decision tree with AUC (0.9287), and SVM polynomial with AUC (0.9258). The AUC values based on the single features are reflected in Tables 1–4.

**Figure 8:**
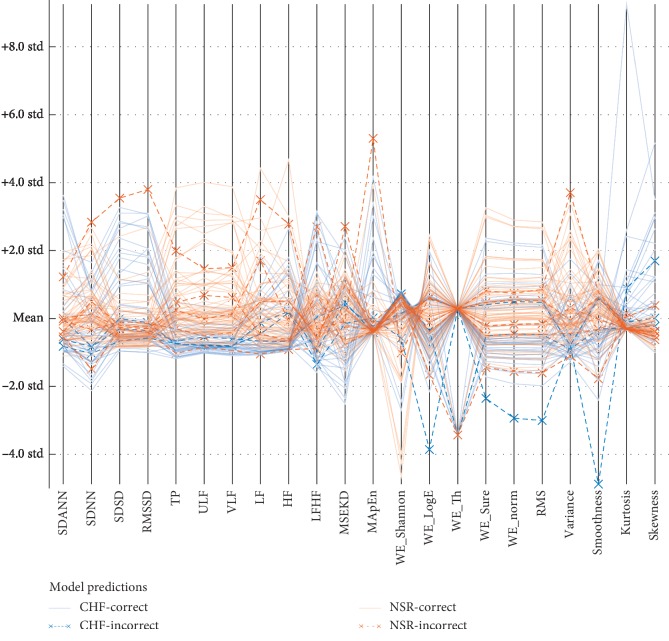
Model prediction to detect heart failure using SVM linear classifier based on multimodal features.

**Figure 9:**
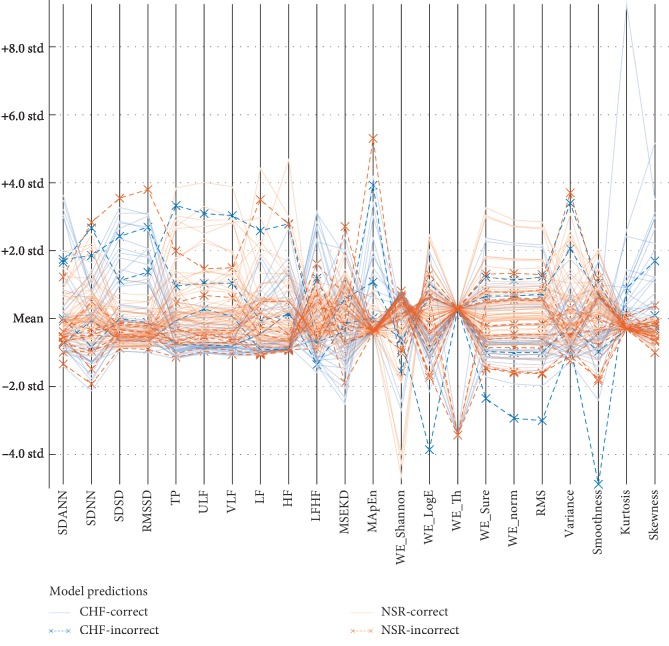
Model prediction to detect heart failure using SVM quadratic classifier based on multimodal features.

In Figures 8 and 9, the blue color denotes the means of CHF subjects and red color denotes the NSR subjects. The lines denote the correctly classified subjects, while *x* denotes the incorrectly classified samples using SVM linear and quadratic kernels. There is a total of 44 CHF subjects and 72 NSR subjects. SVM with linear kernel provides the highest performance with accuracy (93.1%), AUC (0.97) with TP (39), FP (5), FN (3), and TN (69) with less incorrectly classified results, as reflected in Figure 5. Similarly, in Figure 8, SVM quadratic kernel provides accuracy (87.9%), AUC (0.93) with TP (34), FP (10), FN (4), and TN (68) having more incorrectly classified results than SVM linear kernel, as reflected in Figure 9.

We computed the mean ± std from CHF and normal subjects by extracting different time domain, frequency domain, statistical, and entropy-based features as reflected in Table 5. To discriminate these subjects, the *P*-value is reflected in the last column. All the extracted features provided highly significant results to discriminate the CHF subjects from NSR subjects. The significance level is represented by ^*∗∗∗*^*P*-value < ×10^−100^ and > ×10^−50^, ^*∗∗*^*P*-value < ×10^−49^ and > ×10^−25^, and ^*∗*^*P*-value < ×10^−24^ and >0.01. Mostly, the standard features computed gives higher mean values for NSR than CHF subjects. The lowest standard deviations from mean were obtained at SDANN, SDNN, SDSD, RMSSD, MSEKD, MApEn, RMSD, RMSE, variance, smoothness, and skewness. The significance level of ^*∗∗∗*^ was obtained at time domain features (SDNN), entropy-based features (MSEKD, wavelet entropy Shannon, log energy, threshold, sure, and norm), and statistical features (RMS, smoothness, kurtosis). The significance level of ^*∗∗*^ was obtained using time domain features (SDANN), frequency domain features (LFHF ratio), and statistical features (RMS), and the significance level of ^*∗*^ was obtained using time domain features (SDSSD and RMSSD), frequency domain features (TP, ULF, VLF, LF, and HF), entropy-based features (MApEn), and statistical features (Smoothness, kurtosis, and skewness).

## 5. Discussion

The dynamics of heart signals are highly complex and nonlinear in nature. Moreover, the temporal dynamics present in the heart variability based on short-, medium-, and long-term variations can be best captured by extracting time domain features. Moreover, heart rate failure dynamics can also be captured by extracting spectral components which are computed using frequency domain features. The complex dynamics of the dynamical systems can be measured based on structural components and coupling among these components. The complexity degraded when any of the structural/functional components is lost. This loss of complexity is also due to the pathological conditions and aging.

Recently, Kumari et al. [73] in their article concluded that patients with coronary heart disease and diabetes mellitus get significant results in clinical symptoms with improvement in the quality of life. They employed SVM with radial base function kernel and decision support systems to predict the heart rate variability [74]. The results obtained using these methods showed good detection performance. The classification accuracy, sensitivity, and specificity of the SVM and RBF have been found to be high, thus making it a good option for the diagnosis [75].

Based on the varying dynamics of the physiological systems, researchers employed different features of extracting methods. Want et al. [28] extracted discrete wavelet transform (DWT), nonlinear, and multidomain features to detect epileptic seizure and obtained the highest detection accuracy of 99.25%.

Recently, Hussain et al. [76] extracted multimodal features to detect arrhythmia and applied machine learning techniques. Data on CHF and NSR ECG signals were taken from the Beth Israel Deaconess Medical Center (BIDMC) CHF database and the Massachusetts Institute of Technology-Beth Israel Hospital (MIT-BIH) arrhythmia database, respectively. By extracting the frequency domain features (TP, ULF, VLF, LF, HF, and LF/HF), the highest detection performance was obtained using SVM cubic with total accuracy (80.3%) and AUC (0.76). By extracting entropy-based features (sample entropy with KD tree; approximate entropy; and wavelet entropies Shannon, threshold, sure, log energy, and norm), the highest detection accuracy was obtained using SVM medium Gaussian, and fine KNN with total accuracy (100%) and AUC (1.0). Likewise, by extracting time domain and statistical features (SDANN, SDNN, SDSD, RMSSD, RMS, variance, skewness, kurtosis, and smoothness), the highest arrhythmia detection performance was obtained using fine KNN with total accuracy (100%) and AUC (1.0), followed by ensemble bagged tree and subspace discriminant with total accuracy (98.5%) and AUC (0.99 and 1.0, respectively). Moreover, by extracting the entropy-based features, the highest detection performance was obtained using SVM medium Gaussian, fine KNN, and ensemble subspace discriminant with sensitivity (100%), specificity (100%), total accuracy (100%), and AUC (1.0), followed by SVM cubic, medium KNN with sensitivity (98%), specificity (100%), total accuracy (98.5%), and AUC (1.0). Most recently, Tripathy et al. [77] used a similar dataset by extracting time-frequency entropy features and applied a hybrid classifier with mean metric (HCMM); the highest detection accuracy was obtained with sensitivity (98.48%), specificity (99.09%), and accuracy (98.78%). The result reveals that our approach of multimodal features from time domain, frequency domain, statistical, and entropy-based features gives higher detection performance than the feature extracting and classification approach employed by [77] for a similar dataset.

Recently, many studies have been conducted which provided different methods to discriminate CHF patients from normal patients. Isler et al. [78] offered the structure of multistage classifiers in discriminating CHF patients and obtained a specificity of 98.1% and sensitivity of 100%. A recent study [74] investigated the effect of the number of folds in discriminating patients with CHF from normal subjects using five different popular classifiers. It was proved that average performance was enhanced and the variability of performances was decreased when the number of data sections used in the cross-validation method was increased. The highest performance was obtained using KNN with the LOO method having accuracy (80.9%), sensitivity (52.1%), and specificity (96.3%).

Narin et al. [79] investigated the statistical feature selection methods to improve the classifier performances on CHF using HRV analysis. Isler and Kuntalp [80] investigated the effect of heart rate normalization in the classifier performance on CHF patients using HRV analysis. They employed KNN with and without HR normalization by selecting *K* = 1, 3, 5, 7, 9, 11, and 13, with maximum performance of 93.98%. Isler and Kuntalp [81]showed the importance of wavelet-based features in the diagnosis of CHF using HRV signals. They obtained the highest discriminating powers in terms of sensitivity and specificity. The researchers [82] employed different machine learning classifiers such as support vector machine (SVM) with accuracy (81.0%) and random forest with accuracy (81%) to detect and predict the 5 minute preshock data of CHF. Moreover, Sharma et al. [83] applied time-frequency methods for prediction of CHF. Sharma et al. [84] extracted energy and eigenspace to localize and detect myocardial infarction. Similarly, Tripathy et al. [85] employed a novel approach for the prediction of myocardial infarction from ECG signals of multiple electrodes. Moreover, Sharma et al. [86] employed eigenvalue decomposition-based features extracted from HRV signals for automated detection of congestive heart failure.  Table 6 reflects the findings of previous studies.

The present study was aimed to study the dynamics of heart rate variability based on multimodal features by extracting strategy and employing robust machine learning techniques. We have extracted time domain features (to capture short-, medium-, and long-term variations), frequency domain features (to capture spectral components), entropy features (to capture complex dynamics), and applied machine learning classifiers such as support vector machine (SVM) and its kernel, decision tree (DT), K-nearest neighbor (KNN), and ensemble classifiers. Coarse DT gives the highest performance with TPR (85%) and fine DT with PPV (88%). The SVM linear gives performance with TA (93%), TPR (96%), and AUC (0.97), and SVM cubic with TPR (97%), PPV (94%), TA (89.7%), and AUC (0.91). Moreover, the medium KNN gives TPR (99%), PPV (96%), TA (81%), and AUC (0.92). The ensemble method subspace discriminant gives TPR (93%), PPV (89%), TA (91.4%), and AUC (0.96). The results reveal that extracting multimodal features based on time variation, temporal dynamics, and complex dynamics can improve the early detection of heart failure and survival rate.

## 6. Conclusion

Hear rate variability analysis is a noninvasive tool used for assessing the cardiac autonomic control of the nervous system. Various kinds of defects can be detected by analyzing the oscillations between consecutive heart beats. The analysis of HRV is the subject of different clinical studies investigating a wide spectrum of cardiological and non-cardiological diseases and clinical conditions. In other clinical conditions and diseases, a depressed HRV has also been observed in patients suffering from dilated cardiomyopathy, CHF, etc. In this study, we aimed to discriminate the CHF patients from normal subjects after extracting multimodal features. We extracted time domain, frequency domain, statistical, and entropy-based features from CHF and normal subjects and employed the robust machine learning techniques. A 10-fold cross-validation was applied for training and testing data validation. The performance was evaluated in terms of sensitivity, specificity, PPV, NPV, TA, and AUC. We evaluated the CHF detection performance based on single and hybrid features. The highest performance using decision tree was obtained with sensitivity (82%), specificity (82%), and accuracy (81.9%). Using SVM, the highest detection performance was obtained with SVM linear with sensitivity (96%), specificity (89%), and accuracy (93.1%). Moreover, using the ensemble methods, the highest detection performance was obtained using subspace discriminant with sensitivity (93%), specificity (89%), and accuracy (91.4%). The results reveal that by considering temporal, spectral, and nonlinear dynamics, the detection performance of CHF can be very helpful in the early diagnosis and prognosis of heart failure patients.

In the present study, we extracted multimodal features from CHF and NSR subjects and employed machine learning techniques to detect congestive heart failure. In future, we will extract features by considering the clinical information of patients and from the severity level of congestive heart failure classes. We will also apply deep convolutional neural network (CNN) using transfer learning approach for pretrained networks, such as GoogleNet, AlexNet, and Inception V3, as CNN is not feature dependent and is fine-tuned. These directions will provide more detailed and comprehensive studies for further performance improvement.

## Figures and Tables

**Table 1:** CHF detection performance based on time domain features by applying machine learning techniques.

Classifier	TPR (%)	TNR (%)	PPV (%)	NPV (%)	TA (%)	AUC	LB	UP
*Decision tree (DT)*								
Fine	78	77	68	85	77.6	0.73	0.22	0.77
Coarse	89	66	78	81	80.2	0.75	0.11	0.66

*Support vector machine (SVM)*								
Linear	90	73	82	84	83.6	0.92	0.10	0.73
Quadratic	88	66	76	81	79.3	0.84	0.13	0.66
Cubic	85	70	74	82	79.3	0.88	0.15	0.70
Med. Gaussian	89	73	80	84	82.8	0.90	0.11	0.73

*K-nearest neighbor (KNN)*								
Fine	79	59	63	76	71.6	0.69	0.21	0.59
Medium	88	68	77	82	80.2	0.87	0.15	0.75
Cosine	82	80	73	87	81.0	0.83	0.18	0.80

*Ensemble classifiers*								
Bagged tree	85	75	75	85	81.0	0.87	0.15	0.75
Subsp. disc.	96	66	91	82	84.5	0.91	0.04	0.66
RUSBoosted tree	76	68	64	80	73.3	0.81	0.24	0.68

**Table 2:** CHF detection performance based on frequency domain features by applying machine learning techniques.

Classifier	TPR (%)	TNR (%)	PPV (%)	NPV (%)	TA (%)	AUC	LB	UP
*Decision tree (DT)*								
Fine	83	75	73	85	80.2	0.84	0.17	0.75
Coarse	93	64	85	81	81.9	0.81	0.07	0.64

*Support vector machine (SVM)*								
Linear	82	77	72	86	80.2	0.86	0.18	0.77
Quadratic	82	82	73	88	81.9	0.88	0.18	0.82
Cubic	88	68	77	82	80.2	0.83	0.13	0.58
Med. Gaussian	90	77	83	87	85.3	0.90	0.16	0.77

*K-nearest neighbor (KNN)*								
Fine	85	75	75	85	81.0	0.86	0.15	0.75
Medium	89	66	78	81	80.2	0.88	0.11	0.66
Cosine	64	73	55	79	67.2	0.75	0.36	0.73

*Ensemble classifiers*								
Bagged tree	85	77	76	86	81.9	0.88	0.15	0.77
Subsp. disc.	86	70	76	83	80.2	0.85	0.14	0.70
RUSBoosted tree	79	75	69	84	77.6	0.81	0.21	0.75

**Table 3:** CHF detection performance based on statistical features by applying machine learning techniques.

Classifier	TPR (%)	TNR (%)	PPV (%)	NPV (%)	TA (%)	AUC	LB	UP
*Decision tree (DT)*								
Fine	81	68	68	81	75.9	0.77	0.19	0.68
Coarse	86	64	74	79	77.6	0.80	0.14	0.64

*Support vector machine (SVM)*								
Linear	99	55	96	78	81.9	0.80	0.01	0.55
Quadratic	92	64	82	80	81.0	0.84	0.08	0.64
Cubic	85	61	71	78	75.9	0.78	0.15	0.61
Med. Gaussian	90	52	77	76	75.9	0.81	0.10	0.52

*K-nearest neighbor (KNN)*								
Fine	78	55	60	74	69.0	0.66	0.22	0.56
Medium	89	43	70	72	71.6	0.78	0.11	0.43
Cosine	89	48	72	74	73.3	0.78	0.11	0.48

*Ensemble classifiers*								
Bagged tree	85	66	73	80	77.6	0.81	0.15	0.66
Subsp. disc.	99	34	94	71	74.1	0.77	0.01	0.34
RUSBoosted tree	85	66	73	80	77.6	0.79	0.15	0.66

**Table 4:** CHF detection performance based on entropy-based features by applying machine learning techniques.

Classifier	TPR (%)	TNR (%)	PPV (%)	NPV (%)	TA (%)	AUC	LB	UP
*Decision tree (DT)*								
Fine	71	50	51	70	62.9	0.65	0.29	0.50
Coarse	90	36	70	70	69.8	0.69	0.10	0.36

*Support vector machine (SVM)*								
Linear	93	30	72	68	69.0	0.71	0.07	0.30
Quadratic	83	57	68	76	73.3	0.74	0.17	0.57
Cubic	82	52	64	74	70.7	0.73	0.18	0.52
Med. Gaussian	94	30	76	69	69.8	0.75	0.06	0.30

*K-nearest neighbor (KNN)*								
Fine	75	57	58	74	68.1	0.66	0.25	0.57
Medium	85	50	67	73	71.6	0.69	0.15	0.50
Cosine	82	52	64	74	70.7	0.72	0.18	0.52

*Ensemble classifiers*								
Bagged tree	82	57	66	76	72.4	0.78	0.18	0.57
Subsp. disc.	89	39	68	70	69.8	0.71	0.11	0.39
RUSBoosted tree	75	59	59	75	69.0	0.75	0.25	0.59

**Table 5:** Features-based significance level to distinguish the CHF and NSR subjects.

Feature	CHF	NSR	*P*-value
	Mean ± std	Mean ± std	
SDANN	0.010 ± 0.015	0.018 ± 0.008	^*∗∗*^7.68 × 10^−36^
SDNN	0.066 ± 0.032	0.086 ± 0.026	^*∗∗∗*^2.85 × 10^−53^
SDSD	0.056 ± 0.045	0.028 ± 0.018	^*∗*^4.08 × 10^−23^
RMSSD	0.063 ± 0.050	0.035 ± 0.020	^*∗*^1.04 × 10^−24^
TP	347099 ± 316751	858649 ± 563951	^*∗*^1.11 × 10^−24^
ULF	80616 ± 80069	228361 ± 175578	^*∗*^1.5 × 10^−20^
VLF	178871 ± 166993	501651 ± 350518	^*∗*^1.32 × 10^−22^
LF	42353 ± 50725	68217 ± 53525	^*∗*^2.6 × 10^−21^
HF	45257 ± 58350	60419 ± 55206	^*∗*^3.05 × 10^−18^
LFHF	1.442 ± 0.872	1.304 ± 0.389	^*∗∗*^6.55 × 10^−46^
MSEKD	1.370 ± 0.293	1.464 ± 0.179	^*∗∗∗*^2.37 × 10^−93^
MApEn	0.004 ± 0.006	0.0009 ± 0.003	^*∗*^2.07 × 10^−5^
WEShannon	6594 ± 962	6151 ± 1351	^*∗∗∗*^1.38 × 10^−84^
WELogEn	−17460 ± 5702	−14830 ± 5722	^*∗∗∗*^4.62 × 10^−55^
WETh	19999 ± 0.347	19999 ± 0.201	^*∗∗∗*^0
WESure	−11085 ± 2692	−9771 ± 2829	^*∗∗∗*^4.32 × 10^−68^
WENorm	12613 ± 2043	13594 ± 2070	^*∗∗∗*^1.96 × 10^−94^
RMS	0.660 ± 0.096	0.708 ± 0.098	^*∗∗∗*^4 × 10^−99^
Var	0.005 ± 0.005	0.008 ± 0.005	^*∗∗*^4.26 × 10^−27^
Smoothness	0.999 1.08 × 10^−5^	0.999 ± 1.11 × 10^−5^	^*∗∗∗*^0
Kurtosis	40.2 75.4	5.125 ± 7.386	^*∗*^0.000108
Skewness	1.996 ± 2.672	0.264 ± 0.656	^*∗*^8.93 × 10^−7^

**Table 6:** Algorithm comparison of previous studies.

Author	Title of article	Method	Performance
Li et al. [87]	Combining convolutional neural network and distance distribution matrix for identification of congestive heart failure	CNN	TA = 81.9%

Isler and Kuntalp [81]	Combining classical HRV indices with wavelet entropy measures improves to performance in diagnosing congestive heart failure	KNN	ACC = 81.92% Sens = 82.74% Spec = 96.27%

Narin et al. [79]	Investigating the performance improvement of HRV indices in CHF using feature selection methods based on backward elimination and statistical significance	SVM	Sens = 79.33% Spec = 94.47%

Isler and Kuntalp [80]	Heart rate normalization in the analysis of heart rate variability in congestive heart failure	KNN	Sens = 82.72% Spec = 100.0%

Pecchia et al. [88]	Discrimination power of short-term heart rate variability measures for CHF assessment	CART	Sens = 89.75% Spec = 100.0%

Elfadil and Ibrahim [89]	Self-organising neural network approach for identification of patients with congestive heart failure	Spectral NN	ACC = 83.65%

Yang et al. [90]	A heart failure diagnosis model based on SVM	SVM NB CA	TA = 74.42%

Chang et al. [91]	Decision making model for early diagnosis of CHF using rough set and decision tree approaches	RS DT	SEN = 97.53%

Our method	Extraction of multimodal features to predict congestive heart failure (CHF)	DT	Sens = 82% Spec = 82% TA = 81.9%
		SVM linear	Sens = 96% Spec = 89% TA = 93.1%
		EnsembleSubspace discriminant	Sens = 93% Spec = 89% TA = 91.4%

## Data Availability

The data are publicly available on Physionet.
